# Percutaneous cholecystostomy – An option in selected patients with acute cholecystitis

**DOI:** 10.1097/MD.0000000000020101

**Published:** 2020-05-08

**Authors:** Jon Arne Søreide, Anja Fjetland, Kari F. Desserud, Ole Jakob Greve, Lars Fjetland

**Affiliations:** a Department of Gastrointestinal Surgery, Stavanger University Hospital, Stavanger; b Department of Clinical Medicine, University of Bergen, Bergen; c Department of Radiology, Stavanger University Hospital, Stavanger, Norway.

**Keywords:** acute cholecystitis, emergency surgery, laparoscopic cholecystectomy, percutaneous cholecystostomy, severity criteria

## Abstract

While urgent percutaneous cholecystostomy (PC) was introduced as an alternative to acute surgical treatment for acute cholecystitis (AC), the current place of PC in the treatment algorithm for AC is challenged. We evaluate demographics and outcomes of PC in routine clinical practice in a population-based cohort.

Retrospective evaluation of consecutive patients treated with PC for AC between 2000 and 2015. The severity of cholecystitis was graded according to the 2013 Tokyo Guidelines.

One hundred forty-nine patients were included (82; 55% males) (median age of 72.5 years; range, 21–92). The Tokyo Guidelines criteria of 2013 (TG13) severity grade distribution was 4%, 61.7%, and 34.2% for grades I, II, and III, respectively. No difference was observed between males and females with regard to age, American Society of Anesthesiologists (ASA) score, comorbidities, or previous history of cholecystitis. PC was successfully performed in all but 1 patient, and complications were few and minor. Less than half (48.3%) of all patients subsequently received definitive surgical treatment, mostly (83.3%) laparoscopy. No or minor complications were encountered in 58 (80.6%) patients. Operated patients were significantly younger (*P* = <.001) and had lower ASA scores (*P* = .005), less comorbidities (*P* < .001), and had more seldomly a severe grade 3 cholecystitis (*P* < .001) than non-operated patients.

PC is useful in selected patients with AC. However, since only a half of the patients eventually received definitive surgical treatment, a better routine decision-making based on proper criteria may enable an improved allocation of the individual patient for tailored treatment according to the disease severity, the patient's comorbidity burden, and also to the treatment options available at the institution to prevent overutilization of a non-definitive treatment approach. Comprehension of this responsibility should be acknowledged by hospitals with an emergency surgical service, although the clinical decision-making remains a challenge of the responsible surgeon on call.

## 1 Introduction

Acute cholecystitis (AC) is a common cause of acute hospitalization.^[1,2]^ The mean length of stay for AC decreased by 17% (i.e., from 4.7 to 3.9 days; *P* < .05) between 1997 and 2012 in the USA, and the mean hospital charges increased by 195.4% during the same time period.^[2]^ Treatment traditions vary, as shown by the difference in emergency cholecystectomy rates in the USA (52.7%) compared to England (15.7%), with a laparoscopic approach performed in 82.8% of patients in the USA and 37.9% of patients in England.^[3]^

Controversies exist regarding the optimal treatment of AC, and a number of aspects, including the approach (i.e., conservative versus surgery), timing of surgery (acute versus delayed), type of intervention (i.e., cholecystectomy versus partial gallbladder resection or percutaneous cholecystostomy (PC)) have been discussed.^[1,4–10]^ Moreover, the severity of the gallbladder disease as well as the patients’ clinical condition and comorbidities should be considered to accomplish optimal individual decision-making.^[4,5,10,11]^

Since the introduction of ultrasound (US)-guided PC in 1983,^[12]^ this modality has been considered a valuable treatment alternative in the acute situation. This approach was reported as safe and efficient,^[13–17]^ especially in frail or high-risk patients with severe cholecystitis.^[4,11,18]^ However, the true place of PC in the treatment algorithm for AC is still debated.^[18–21]^

This study was performed to evaluate patients’ clinical patterns, technical success rates, and outcomes after treatment with PC in a cohort of consecutive unselected patients with AC.

## 2 Methods

Stavanger University Hospital serves as the only hospital for an urban and rural catchment area comprising a population of around 380,000 people.

Due to our treatment traditions patients with AC were mostly treated conservatively (i.e., typically with antibiotics). In elderly and frail patients with significant comorbidities and a suggested diagnosis of severe AC, PC was considered early in the course of disease, as decided by the responsible surgeon. Moreover, PC would be contemplated when a detrimental clinical course of disease was encountered in spite of optimal conservative treatment.^[22]^

### 2.1 Patients

The patients were identified based on information from the diagnosis and procedure codes from the electronic hospital records administrative systems.

AC was diagnosed based on clinical symptoms and signs (right upper quadrant tenderness and a positive Murphy sign), supported by laboratory data (elevated C-reactive protein (CRP) levels and leukocytosis), and confirmed by imaging, mostly by US but sometimes by computed tomography (CT).

Patients who underwent PC for reasons other than an AC were excluded from this study.

### 2.2 Diagnosis and classification of AC

Acute *calculous* cholecystitis (ACC) was diagnosed based on symptoms, clinical findings, and imaging as described by others.^[23]^ Acute *acalculous* cholecystitis (AAC) is characterized by severe gallbladder inflammation without cystic duct obstruction due to gallstones.^[24]^

The cholecystitis severity grading (grade 1–3) for this study was performed *retrospectively* according to the Tokyo Guidelines criteria of 2013 (TG13).^[25,26]^

### 2.3 PC

A radiologist trained in US-guided interventions performed the PC under conscious sedation and local anesthetics. The gallbladder was directly punctured under sonographic guidance, preferably through a small brim of liver.^[27,28]^ A positive clinical response was suggested when a resolution of clinical symptoms and signs was encountered, with a decrease or disappearance in pain, fever, and leukocytosis or CRP levels, along with the absence of adverse events related to PC or catheter removal.^[22]^

A follow-up fluoroscopic biliary study with contrast was mostly performed 3 to 5 days after the PC to evaluate if contrast could pass the cystic duct and to assess if contrast reached the duodenum. The drain was clamped and removed the next day if free contrast passage was confirmed and clinical condition was improving. The catheter was left in place if no contrast passage was visualized. Patients in good general clinical condition were discharged with the drain in place, and a secondary cholangiography was performed after another 2 to 3 weeks. In most cases, the catheter was removed at this time.

At a surgical out-patient clinic consultation 4 to 6 weeks after PC, indications for an elective cholecystectomy was discussed with the patient, and scheduled for at least 2 to 3 months after the index admission

### 2.4 Complications

Postoperative complications (i.e., related to the delayed cholecystectomy) were categorized according to the Clavien–Dindo classification system.^[29,30]^ Class 0 to II include no or minor complications, and class ≥III include major complications.

### 2.5 Ethical approval and consent to participate

All procedures performed were in accordance with the ethical standards of the institutional and/or national research committee and with the 1964 Helsinki declaration and its later amendments or comparable ethical standards.

The study was approved by the Regional Committee for Medical and Health Research Ethics, University of Bergen, Bergen, Norway (ref. no. 2015/1496/REK-Vest). This approval includes the consent for publication.

### 2.6 Statistics

IBM Statistics SPSS for Mac v.25 was used for statistical analysis. Results are reported as the median (range) for continuous variables and proportions (percentages) for categorical variables. A non-normal distribution was suggested for the continuous variables. The Mann–Whitney *U* test was used to analyze differences between continuous variables, and the chi-square test was used to compare categorical variables. A *P*-value <.050 was regarded as statistically significant.

The results are reported according to the STROBE criteria for observational studies.^[31]^

## 3 Results

Between 2000 and 2015, a total of 149 patients (82; 55% males; median age of 72.5 (range, 21–92) years) with AC were treated with PC. Demographics and clinical characteristics are displayed in Table 1. No significant differences between males and females were observed with regard to age, ASA score, comorbidity/Charlson comorbidity index (CCI), or previously reported history of bile colic or cholecystitis. While the AC severity grades (TG13 criteria^[25]^) were similarly distributed between sexes, *acalculous* cholecystitis was significantly (*P* = .006) more common in males (19.5%) than in females (4.5%) (Table 1).

**Table 1:**
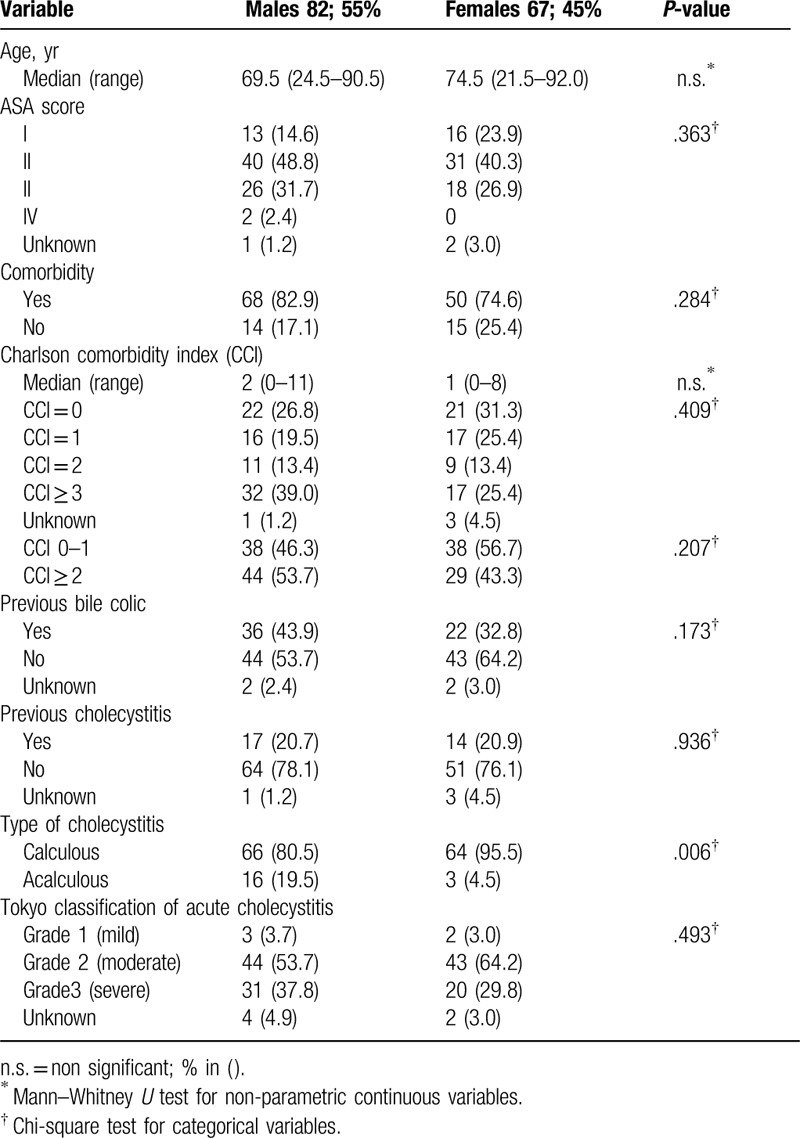
Demographics and clinical characteristics of 149 patients according to sex.

**Figure 1:**
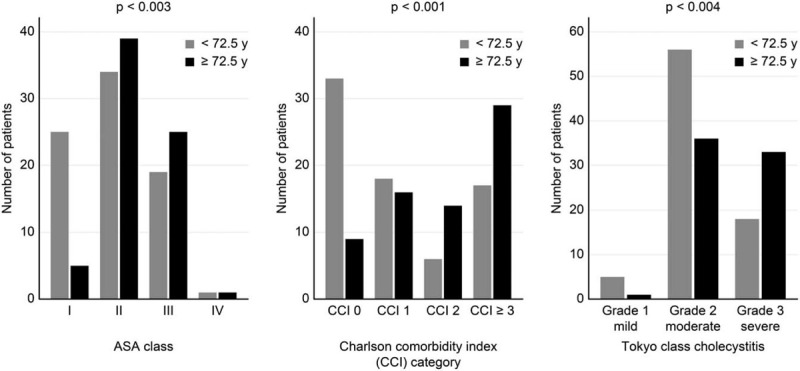
Distribution of ASA class, Charlson comorbidity index (CCI), and severity of acute cholecystitis (TG13) in younger (i.e., <median age of 72.5 yr) and older (≥72.5 yr) patients.

**Figure 2:**
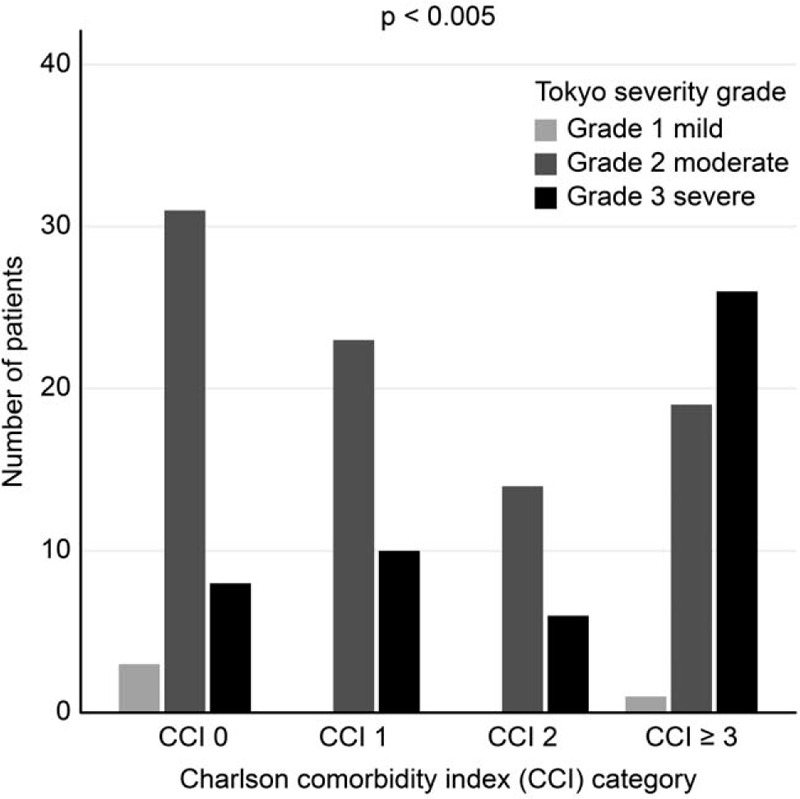
Distribution of severity grade (TG13) by CCI groups.

When younger patients (i.e., median <72.5 years of age) were compared with older patients, no differences were found with regard to sex distribution or history of previous biliary colic or AC. In contrast, the ASA score distribution (*P* < .003), CCI (*P* < .001), and TG13 severity grades (*P* = .004) differed significantly between age groups (Fig. 1). Moreover, as shown in Figure 2, the severity of AC at admission to the hospital differed significantly (*P* < .005) according to the comorbidity category of the patients, with the highest proportion of patients with severe cholecystitis (i.e., TG13 grade 3) found in patients with the greatest comorbidity burden (i.e., CCI ≥ 3).

Duration symptoms before admission to the hospital was median 2 days. AC occurred as an in-hospital complication in 12 (8%) patients admitted for other medical conditions. Five of these 12 patients had acalculous cholecystitis, which was a significantly higher proportion than that among patients (14/137; 10.2%) diagnosed with acalculous cholecystitis at admission to the hospital (*P* < .001).

US, either alone or in combination with other biliary imaging modalities (i.e., mostly CT), was employed in more than 70% of the patients; 25% underwent CT alone, and very few had magnetic tomography (MT) alone or in combination with US.

Half of the PC procedures (51%) were completed during normal working hours. Insertion of the drain was successful in all but 1 patient due to lack of cooperation. Typically, the transhepatic route was used, and this approach was confirmed in 75 (50.2%) of the patients. Direct puncture of the gallbladder was performed in 18 (12.1%) of the patients. Reliable information to confirm the exact catheter route was not available in the remaining patients.

Except for transitory and moderate pain in some patients, no severe adverse advents after PC were noted. Minor bleeding was observed in 2 patients, and 1 patient suffered from transient pain-related dyspnea for a few hours.

A pericholecystic abscess was found in 21 (14%) of the patients. A positive bacterial culture, either from blood or bile samples, was obtained in 70 (47%) of the patients. *Escherichia coli* was the most frequently encountered bacteria, either alone or in combination with other bacteria.

Scheduled follow-up cholangiography was performed at a median of 5 days after insertion of the drain. Free passage of contrast to the duodenum was confirmed in 73 (49%) patients. Of note, 33 patients (22%) had drains removed unintentionally in the ward, but only 13 of these patients (i.e., less than one-third) required repeated PC. In the remaining 20 patients, reintervention was not recommended due to their improving clinical conditions. Thus, in 27 patients (18%), postprocedural follow-up cholangiography was not possible, mostly (20/27 = 74%) due to unintentional removal of the drain without replacement.

The 30-day mortality rate after admission was 5.4% (8 patients) including 4 related to biliary sepsis and 1 to biliary severe pancreatitis. The remaining 3 frail patients died from other medical causes (i.e., cardiovascular or pulmonary incidents). A cumulative 90-day mortality rate of 7.4% (11/149) was observed. No deaths were related to the PC procedure itself.

Definitive surgical treatment was eventually performed in 72 patients (48.3%), including 35 (48.6%) females. As seen in Table 2, the operated patients were younger (*P* < .001), had a lower ASA score (*P* = .005), and a lower CCI (*P* < .001). Moreover, the cholecystitis severity grade was lower in patients who underwent subsequent surgery (*P* < .001).

**Table 2:**
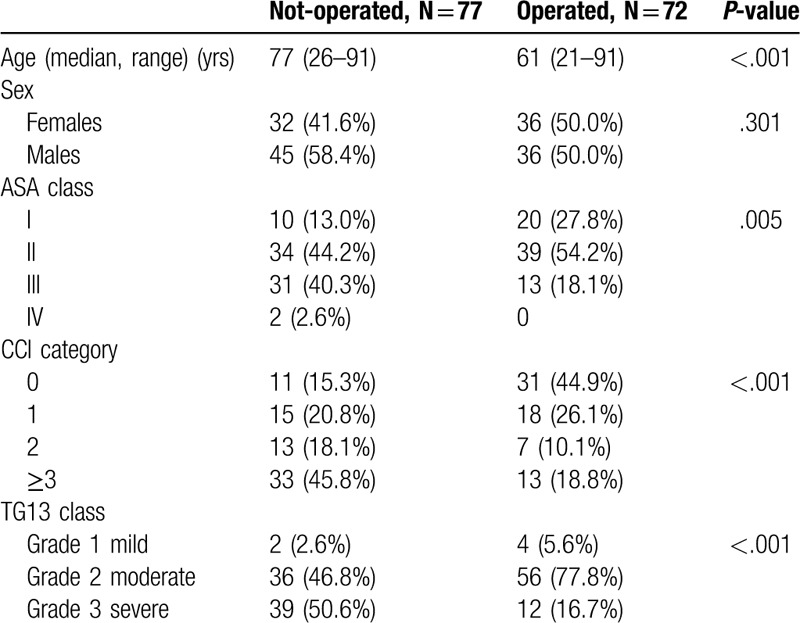
Clinical characteristics of operated and non-operated patients.

Surgical treatment was employed in 7 patients (9.9%), and 32 patients (50%), within 30 days and 3 months, respectively. And 95% of the surgically treated patients had been operated within a year from the index admission. However, around one-third (24/72) of the operated patients had a new AC episode with readmission to the hospital before definitive surgery could be completed.

A laparoscopic approach was used in 60 patients (60/72 = 83.3%), including 57 cholecystectomies and 3 partial gallbladder resections. Conversion to open cholecystectomy was necessary in 3 patients (4.3%) due to perioperative challenges related to extensive inflammation. No or minor postoperative complications (i.e., Clavien–Dindo class I–II) were recorded in 54 (80.6%) of the operated patients. No bile duct injury was recorded.

**Figure 3:**
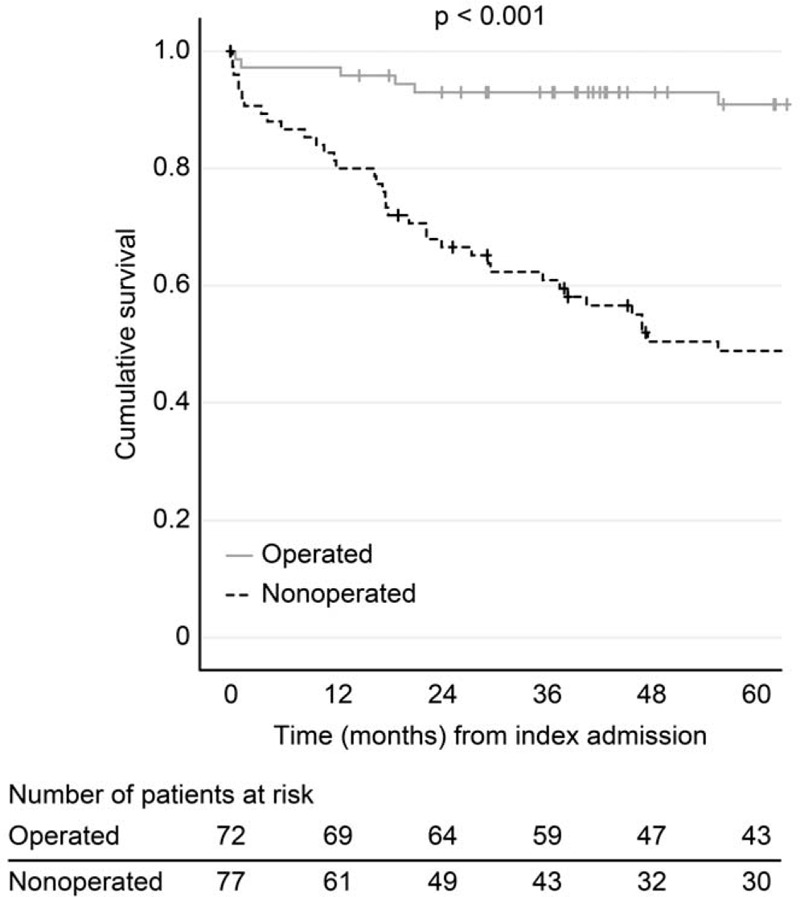
Survival of the patients (Kaplan–Meier plot) according to whether definitive surgical treatment was completed or not.

During a median follow-up of 56 months (interquartile range (IQR), 26–100 months), 62 patients (42%) eventually died, and 3 were lost to follow-up due to emigration (Fig. 3). However, during the first year after the index admission, 18.2% (14/77) of the non-operated patients died of causes unrelated to gallbladder disease compared to 2.7% (2/72) of the surgically treated patients (*P* = .006). Thus, PC was the final treatment in 77 patients (52%), of whom 15 patients (19.5%) were eventually readmitted with a new episode of AC. Altogether, 39 (26.2%) patients (i.e., 24 (33.3%) of the operated and 15 (19.5%) of the non-operated patients) had at least 1 readmittance to the hospital for cholecystitis-related complaints or symptoms. The cumulative death rates for the *non-operated patients* were 18.2%, 31.2%, and 37.7%, at 1, 2, and 3 years after the index admission, respectively.

## 4 Discussion

The majority of patients responded properly to the initial treatment with PC. No procedure-related deaths were observed, although 3 patients died during the index admission because of severe infection. Thus, as also reported by others,^[11,32–35]^ PC is regarded as a safe procedure of selected patients with AC.

Catheter dislodgement was observed in 22% of our patients. Of note, less than half (39%) of these patients needed a new percutaneous drain. While complications were rare in this study, a wide range of uncommon complications including hemorrhage, vagal reactions, bile peritonitis, sepsis, pneumothorax, bowel perforation, or secondary infection have been reported.^[27,28]^

Subsequent cholecystectomy, either early or delayed, is considered an important part of the definitive treatment of patients with AC.^[1,5,10]^ While recent studies^[9,36,37]^ have provided convincing support for early surgical treatment in patients with AC, surgery is often postponed in multimorbid or frail patients in particular, either due to different practices,^[3]^ opinions,^[38]^ or experience with subgroups of patients.^[22,39]^ Thus, more than 50%, as recently summarized by Stanek et al,^[40]^ never undergo definitive surgery, which is in line with 48.3% surgically treated patients observed in this study. Moreover, in a large population-based study by Lu et al,^[21]^ only 36.4% of the 11,184 patients who were treated with PC eventually underwent subsequent cholecystectomy, which parallels 31.4% as reported recently from Canada.^[41]^

Among the 72 (48.3%) patients who underwent surgery in this study, about one-third (24 patients) were readmitted with a new episode of AC before surgery could be completed. Moreover, within a year from the index admittance, 2.7% (2/72) of the surgically treated patients had died in contrast to 18.2% (14/77) of the non-operated patients (*P* < .01). Notably, patients who died were older and had significant comorbidities. Thus, this survival difference is likely explained by patient selection.

In a recent nationwide randomized-controlled trial (RCT) from the Netherlands (CHOCOLATE-study) on high-risk patients (i.e., APACHE II ≥ 7) with AC, laparoscopic cholecystectomy was compared with percutaneous catheter drainage.^[42]^ The authors concluded that both from a clinical and economical point of view, laparoscopic cholecystectomy should be the preferred strategy compared with PC.^[42]^ However, as with all RCTs, a number of exclusion criteria distorted the study population, and direct comparison with unselected consecutive routine patients (i.e., our study population) is challenging.

We used the previous TG13 for our retrospective classification of disease severity. The diagnostic criteria and classification of AC remained the same in the most recent 2018 version of these guidelines.^[43,44]^ Of note, indications for PC for patients with AC were not based on strict predefined criteria in our routine practice. By reviewing our unselected patient cohort, it seems obvious that rather than using criteria for the severity of AC, the indications for PC were mainly guided by the surgeon's perception of a compromised patient with an obvious comorbidity, thus regarded at an increased risk of acute surgery. This approach is also in accordance with others, as recently reviewed by Dimou and Riall.^[45]^

The duration of gallbladder drainage varies remarkably between 2 and 193 days in different studies.^[28,46]^ Our own routine aims at an early catheter cholangiography (i.e., after 3–5 days), to prepare for an early removal of the drain when the clinical conditions allowed. We achieved this goal in approximately half of the patients. Some authors claim that the drain should routinely remain for at least 2 to 4 weeks to ascertain proper drainage.^[28,45,46]^ As summarized in a recent systematic review, at the moment there is no evidence regarding whether the duration of the PC tube may affect outcomes.^[47]^

As shown in Figure 1, patients <72.5 years (i.e., the median age) had less compromised physical condition (i.e., lower ASA score) and comorbidities (i.e., lower CCI) than older patients, and often mild or moderate AC. Indications for PC are outlined in a recent international consensus report^[4]^ and described in the TG13^[26]^ and the most recent Tokyo Guidelines criteria of 2018 (TG18).^[43]^ Based on the retrospective review of the patients, and evaluation of the severity of the AC, we now think that PC may have been overutilized in some patients in this study.

A strength of this study is the population-based unselected study population. Referral bias is unlikely. The retrospective classification of the severity of cholecystitis according to the TG criteria was performed by experienced clinicians and radiologists who reviewed available clinical information and recorded imaging files. A complete follow-up was possible by collecting appropriate information from the hospital records, which are linked to Statistics Norway (www.ssb.no), to provide the date of death in deceased patients.

The retrospective design of this study is an obvious limitation. In spite of this, data completeness was good, which allows for a reliable evaluation of the routine clinical use of PC in patients suggested to have severe AC during that time period.

## 5 Conclusions

Although feasible and safe, and useful in a select group of patients, PC should not be overutilized when alternative and more definitive surgical treatment approaches are indicated and possible. Proper clinical decision-making should be based on scientific evidence. In routine practice, decision-making should rest on reasonable criteria that take into consideration both the individual patient's condition and comorbidity burden as well as the disease severity. All hospitals with an emergency surgical service that has the clinical treatment responsibility of patients with acute cholecystitis should have a masterplan for the management of these often frail and elderly patients. Mutual collaboration with other specialists, including radiologists, intensivists and others, is recommended. However, the clinical decision-making of the individual patient with acute cholecystitis remains a challenge primarily for the responsible surgeon involved.

## Author contributions

**Acquisition of data:** JAS, AF, KFD, OJG, LF.

**Analysis and interpretation of data:** JAS, AF, LF.

**Critical revision:** JAS, AF, KFD, OJG, LF.

**Drafting of manuscript:** JAS, AF.

**Study conception and design:** JAS.
